# An *In Vitro* Model for Lewy Body-Like Hyaline Inclusion/Astrocytic Hyaline Inclusion: Induction by ER Stress with an ALS-Linked SOD1 Mutation

**DOI:** 10.1371/journal.pone.0001030

**Published:** 2007-10-10

**Authors:** Satoru Yamagishi, Yoshihisa Koyama, Taiichi Katayama, Manabu Taniguchi, Junichi Hitomi, Masaaki Kato, Masashi Aoki, Yasuto Itoyama, Shinsuke Kato, Masaya Tohyama

**Affiliations:** 1 Department of Anatomy and Neuroscience, Graduate School of Medicine, Osaka University, Suita, Osaka, Japan; 2 The 21st Century Center of Excellence Program, Graduate School of Medicine, Osaka University, Suita, Osaka, Japan; 3 Department of Neurology, Tohoku University School of Medicine, Sendai, Japan; 4 Department of Neuropathology, Institute of Neurological Sciences, Faculty of Medicine, Tottori University, Yonago, Japan; Emory University, United States of America

## Abstract

Neuronal Lewy body-like hyaline inclusions (LBHI) and astrocytic hyaline inclusions (Ast-HI) containing mutant Cu/Zn superoxide dismutase 1 (SOD1) are morphological hallmarks of familial amyotrophic lateral sclerosis (FALS) associated with mutant SOD1. However, the mechanisms by which mutant SOD1 contributes to formation of LBHI/Ast-HI in FALS remain poorly defined. Here, we report induction of LBHI/Ast-HI-like hyaline inclusions (LHIs) *in vitro* by ER stress in neuroblastoma cells. These LHI closely resemble LBHI/Ast-HI in patients with SOD1-linked FALS. LHI and LBHI/Ast-HI share the following features: 1) eosinophilic staining with a pale core, 2) SOD1, ubiquitin and ER resident protein (KDEL) positivity and 3) the presence of approximately 15–25 nm granule-coated fibrils, which are morphological hallmark of mutant SOD1-linked FALS. Moreover, in spinal cord neurons of L84V SOD1 transgenic mice at presymptomatic stage, we observed aberrant aggregation of ER and numerous free ribosomes associated with abnormal inclusion-like structures, presumably early stage neuronal LBHI. We conclude that the LBHI/Ast-HI seen in human patients with mutant SOD1-linked FALS may arise from ER dysfunction.

## Introduction

Amyotrophic lateral sclerosis (ALS) is a progressive neurodegenerative disorder in which both upper and lower motor neurons begin to degenerate in middle-aged persons. About 10% of ALS patients demonstrate autosomal dominant inheritance of this disease, a disorder known as familial ALS (FALS) [1]–[6]. About 20% of FALS cases are associated with mutations of the Cu/Zn-superoxide dismutase (SOD1) gene [7]. SOD1 is an abundant protein of approximately 153 amino acids that accounts for approximately 1% of total cytosolic protein. More than 100 different SOD1 mutations have been reported as risk factors in association with FALS.

The endoplasmic reticulum (ER) is responsible for the synthesis, initial post-translational modification, and proper folding of proteins, as well as for their sorting export and delivery to appropriate cellular destinations. A variety of conditions, such as loss of the intraluminal oxidative environment or loss of calcium homeostasis, can cause accumulation of misfolded proteins in the ER. To cope with such accumulation, there are three possible responses in eukaryotes. The first response is known as the unfolded protein response (UPR), in which IRE1α and ATF6 recognize aberrant proteins and increase the expression of ER-resident chaperones such as GRP78/BiP and GRP94 to promote proper protein folding [8], [9]. The second response involves suppression of translation mediated by the serine/threonine kinase PERK, which phosphorylates and inactivates the translation initiation factor eIF-2α to reduce the production of misfolded proteins [10], [11]. The third response is ER-associated degradation (ERAD), in which misfolded proteins are expelled from the ER and targeted for degradation by cytoplasmic proteasomes [12], [13]. Although these three protective responses can transiently control the accumulation of misfolded proteins within the ER, they can be overcome by sustained ‘ER stress’ [14]–[16]. ‘ER stress’ is involved in neuronal death and various neurodegenerative disorders, such as Charcot-Marie-Tooth disease, and is especially related to inclusion body diseases such as Alzheimer's disease, Parkinson's disease, Huntington's disease and ALS [17]–[23].

Histopathologic studies have revealed that neuronal Lewy body-like hyaline inclusions (LBHI) and astrocytic hyaline inclusions (Ast-HI), are morphological hallmarks of mutant SOD1-linked FALS [24]. Neuronal LBHI and Ast-HI are ultrastructually identical and share various features, with both consisting of 15–25 nm granule-coated fibrils, both showing immunoreactivity for SOD1, ubiquitin, and copper chaperone for SOD (CCS), and both appearing late in the course of the disease (i.e. at ∼10 to 30 years of age in humans [24]–[27]). Recently, Wate et al. reported that neuronal LBHI are immunoreactive for GRP78/BiP, a component of the UPR cellular response to ER stress [28].

In the present study, we show that ER stress in a neuroblastoma line expressing mutant SOD1 can provoke SOD1 aggregation in ER and formation of LBHI/Ast-HI-like hyaline inclusion bodies (LHIs), which show SOD1, ubiquitin, GRP78/BiP and ER resident protein (KDEL) immunopositivity similar to the shared cytopathological features of LBHI and Ast-HI. Induced neuroblastoma LHI furthermore consisted of 15–25 nm granule-coated fibrils, a hallmark of mutant SOD1-linked FALS, raising the possibility that these acutely induced aggregations represent a precursor to LBHI/Ast-HI seen in advanced FALS. In support of this possibility, we observe abnormal ER and numerous free ribosomes aggregated in the peri-nuclear region neuroblastoma cells expressing L84V SOD1 under ER stress condition and in spinal cord neurons in presymptomatic transgenic mice expressing L84V SOD1. Taken together, these findings suggest a model for early events in FALS cellular pathology, in which ER stress promotes the aggregation of mutant SOD1 and is involved in the development of LBHI/Ast-HI in patients with mutant SOD1 linked FALS.

## Result

### Aggregation and ubiquitination of mutant SOD1 under ER stress

**Figure 1:**
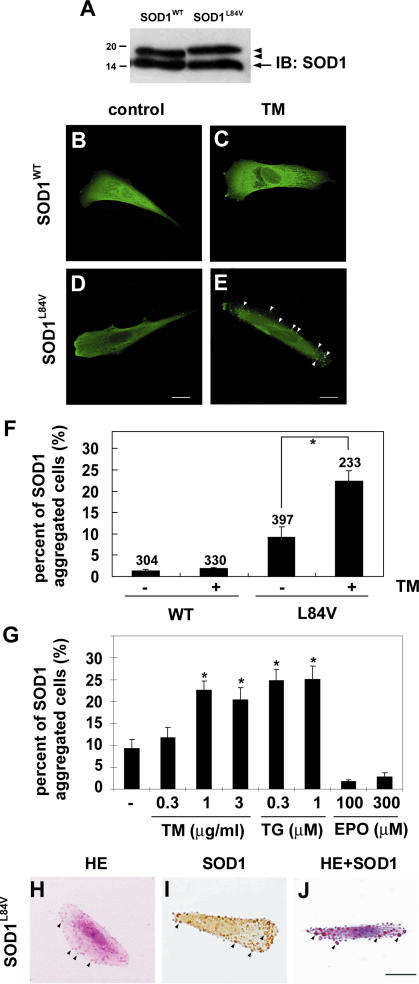
Eosinophilic aggregates of L84V SOD1 are induced by ER stress. (A) Western blotting analysis of the expression of SOD1 in SK-N-SH cells, which stably expressed FLAG tagged wild-type SOD1 or L84V mutant SOD1. Arrowheads and arrow indicate exogenous and endogenous SOD1, respectively. (B–D) Immunofluorescent analysis of SOD1 aggregates in SK-N-SH cells expressing wild-type SOD1 (B, C) or L84V SOD1 (D, E). Cells were incubated under control conditions (B, D) or with 1 µg/ml tunicamycin (C, E) for 24 h, and then were fixed and stained with an anti-SOD1 antibody. Tunicamycin induced aggregates of SOD1 (arrowheads) in L84V SOD1-expressing cells, but not in wild-type SOD1-expressing cells. Scale bar = 20 µm. (F) Quantification of (B-D). After the staining the cells with SOD1 aggregates were counted and scored. Numbers indicate the amounts of total counted cells. Asterisks show a significant difference from control, *p<0.001. (G) SOD1 aggregates induced by tunicamycin and thapsigargin, but not by etoposide. SK-N-SH cells expressing L84V SOD1 were exposed to 0.3, 1 and 3 µg/ml tunicamycin, 0.3 and 1 µΜ thapsigargin and 100 and 300 µΜ etoposide. Asterisks show a significant difference from control, *p<0.001. (H-J) Eosinophilic SOD1 aggregates induced by tunicamycin. Cells were treated as described in (E) and then stained with HE (H), anti-SOD1 antibody (I), or both (J). Scale bar = 20 µm.

To identify conditions which lead to the aggregation of mutant SOD1, we generated SK-N-SH human neuroblastoma cell lines that stably expressed FLAG-tagged human SOD1 encoding a leucine to valine substitution mutation (L84V) associated with FALS [29]. Western blot analysis confirmed that expression of endogenous and exogenous SOD1 was equal in the cell line (Fig. 1A). Reports that neuronal LBHI contain GRP78/BiP, an ER resident component of the UPR response, suggested that ER stress might be a factor in the aggregation of mutant SOD1 [28]. We therefore examined localization of wild-type and mutant SOD1 under normal conditions and under conditions of ER stress (Figure 1). Under normal conditions, wild-type and L84V SOD1 were distributed through the cytosol (Fig. 1B and D). However, following treatment with tunicamycin, an inhibitor of N-glycosylation which causes ER stress, small SOD1-positive aggregates (up to 3 µm in diameter) were seen in L84V SOD1-expressing cells (22.3%, p<0.001; Fig. 1E and F). A much smaller percentage of wild-type SOD1 expressing cells (2.9%, n.s.) showed non-inducible SOD1 aggregation (Fig. 1C and F). To confirm whether ER stress is required for the aggregation of SOD1, we compared tunicamycin and thapsigargin as ER stress inducers with etoposide as a non-ER stress inducer (causing DNA damage). Exposure to 1 and 3 µg/ml tunicamycin (21.1% and 17.5%, respectively) or 0.3 and 1 µM thapsigargin (27.0% and 27.2%, respectively) significantly increased the number of cells containing SOD1 aggregates, in L84V SOD1 expressing neuroblastoma cells. Treatment with 100 and 300 µM etoposide did not lead to a significant increase in aggregates (Fig. 1G). Thus mutant SOD1 forms aggregates following treatments provoking ER stress, but not following treatment causing damage to the nucleus.

Since the SOD1-positive inclusions of FALS patients are known to be eosinophilic [26], we performed hematoxylin-eosin (HE) and anti-SOD1 antibody staining to determine whether the aggregates induced in the neuroblastoma line were also eosinophilic. Figures 1H–J show that the aggregates induced by tunicamycin treatment were positive for both eosin and SOD1.

**Figure 2:**
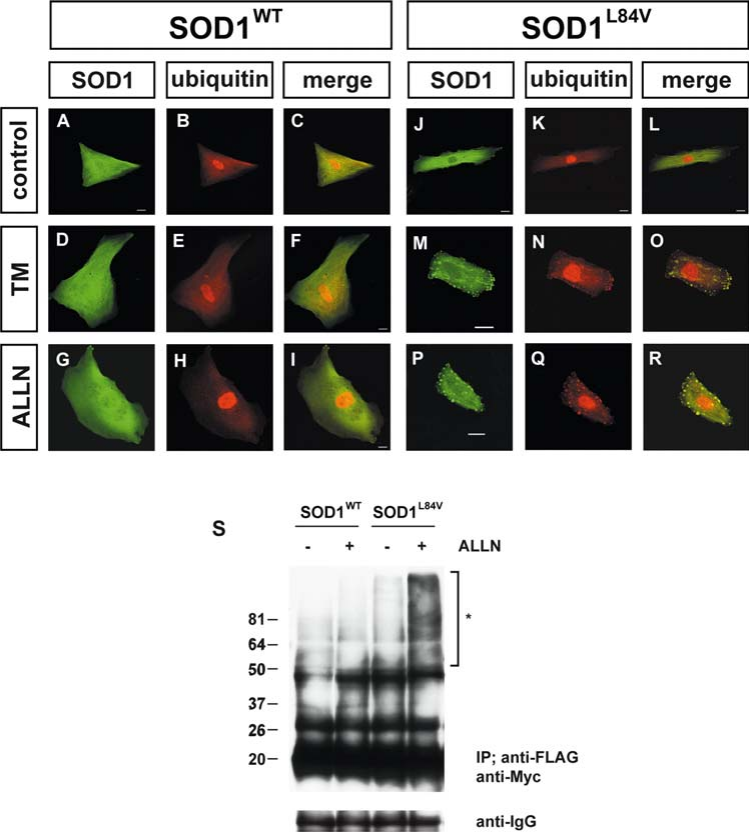
Ubiquitination of mutant SOD1 aggregates. (A–R) Colocalization assay with SOD1 and ubiquitin. SK-N-SH cells expressing wild-type SOD1 (A–I) or L84V SOD1 (J–R) were incubated with 1 µg/ml of tunicamycin (D–F, M–O), 4 µg/ml of ALLN (G–I, P–R), or no agents (A–C, J–L) for 24 h. Then the cells were fixed and stained with anti-SOD1 antibody (green; A, D, G, J, M, P) or anti-ubiquitin antibody (red; B, E, H, K, N, Q). Arrows indicate colocalization of SOD1 aggregates and ubiquitin. Scale bar = 20 µm. (S) Co-immunoprecipitation assay utilizing ubiquitin. SK-N-SH cells stably expressing wild-type and L84V SOD1 were transfected with a myc-tagged ubiquitin expression vector. After incubation with or without ALLN, cell lysates were prepared and assayed with anti-myc antibody of the immunoprecipitant with anti-FLAG antibody. Asterisk shows an ubiquitinated ladder that appeared after ALLN treatment of L84V SOD1-expressing cells. IgG bands are shown as loading controls.

In patients with mutant SOD1-linked FALS, SOD1-positive aggregates are reported to be ubiquitinated by RING finger-type E3 ubiquitin ligases such as dorfin [30]–[33]. To investigate whether the SOD1 aggregates induced by ER stress were ubiquitinated, we performed double immunostaining with anti-SOD1 and anti-ubiquitin antibodies (Fig. 2 A–R). After treatment with either tunicamycin or ALLN, a specific proteasome inhibitor, wild-type and L84V SOD1-expressing cells were immunostained with anti-SOD1 and anti-ubiquitin antibodies. As a result, mutant SOD1 aggregates induced by either tunicamycin or ALLN were clearly colocalized with ubiquitin, suggesting the SOD1 were ubiquitinated. To further examine the ubiquitination of the mutant SOD1, a co-immunoprecipitation assay utilizing ubiquitin was performed (Fig. 2S). As expected, L84V SOD1-expressing cells showed a positive ubiquitin ladder after ALLN treatment, but wild-type SOD1-expressing cells did not.

### Aggregates of SOD1 show positive localization to the ER, but not to the mitochondria, lysosomes, or Golgi apparatus

**Figure 3:**
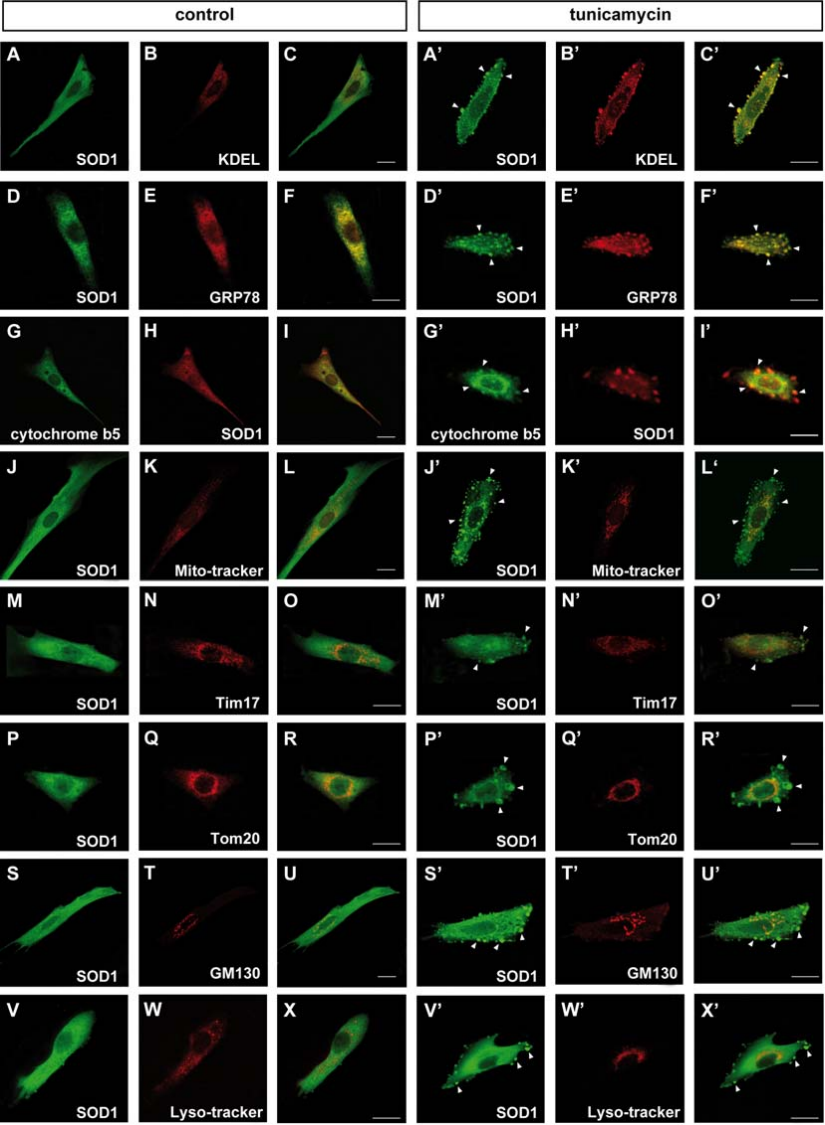
Positive translocation of SOD1 aggregates to ER, but not to the mitochondria, Golgi apparatus, or lysosomes. (A–I, A′–I′) Stress-dependent localization of SOD1 to the ER. L84V SOD1-expressing SK-N-SH cells were incubated for 24 h without (A–I) or with 1 µg/ml of tunicamycin (A′–I′). Then the cells were fixed and stained using an anti-SOD1 antibody (green; A, D, A′, D′) and an anti-KDEL antibody (red; B, B′) or an anti-GRP78/BiP antibody (red; E, E′). GFP-cytochrome b5 were transfected to the cells and stained with anti-GFP (green; G, G′) and anti-SOD1 (red; H, H′) antibodies. Merged images (C, F, I, C′ F′, I′). The aggregates of SOD1 (arrowheads) are positive for KDEL, GRP78/BiP and cytochrome b5. (J–R, J′–R′) Analysis of SOD1 localization to the mitochondria. L84V SOD1-expressing SK-N-SH cells were treated as described in above. The locations of the mitochondria and SOD1 were visualized in L84V SOD1-expressing SK-N-SH cells using 100 nM Mito-tracker (red; K, K′), an anti-Tim17 antibody (red; N, N′) or an anti-Tom20 antibody (red; Q, Q′) and an anti-SOD1 antibody (green; J, M, P, J′, M′, P′). Merged images (L, O, R, L′, O′, R′). (S–U, S′–U′) Investigation of SOD1 localization to the Golgi apparatus. L84V SOD1-expressing SK-N-SH cells were treated as described in above. Then the cells were stained with anti-SOD1 antibody (green; S, S′) and anti-GM130 antibody (red; T, T′). Merged images (U, U′). (V-X, V′-X′) Analysis of the localization of SOD1 to the lysosomes. A GFP-tagged L84V SOD1 vector was transfected into L84V SOD1-expressing SK-N-SH cells. After 24 h of incubation with 1 µg/ml of tunicamycin, the cells were incubated for a further 30 min with 100 nM Lyso-tracker (red; W, W′) to visualize the lysosomes. GFP channel (V, V′) Merged images (X, X′). Scale bars = 20 µm. Arrowheads indicate aggregated SOD1.

Under normal conditions, SOD1 is diffusely distributed throughout the cytoplasm. In contrast, under the pathological condition, SOD1 aggregates are associated with specific organelles such as the mitochondria and/or ER [34]–[37]. Since the tunicamycin-induced aggregates of mutant SOD1 were localized to the central and peripheral regions of the cytoplasm (Fig. 1E, H–J), we investigated the subcellular localization of these aggregates with organelle specific markers. Confocal microscopy analysis clearly showed colocalization of SOD1 and an ER retention signal (KDEL) containing protein and GRP78/BiP, suggesting SOD1 localization in ER (Fig. 3A–F, A′–F′). In order to confirm the SOD1 colocalization with ER, we utilized GFP conjugated cytochrome b5, a typical C-terminal anchored ER membrane protein. As expected, SOD1 showed the positive staining with cytochrome b5, indicating mutant SOD1 localization to ER (Fig. 3G–I, G′–I′). In the absence of stress, ER was located to the perinuclear region. However, treatment with tunicamycin seemed to cause its relocation to an abnormal region near the cell periphery. The aberrant distribution of ER following tunicamycin treatment was not observed in cells expressing wild type SOD1 (Fig. S1C′, F′ and I′). These results suggest deterioration of ER function and localization due to aggregation of mutant SOD1.

In light of previous reports identifying mutant SOD1 colocalization to the mitochondria [34], [35], [37], we also examined the potential colocalization of mutant SOD1 with mitochondria. In contrast to the results with markers for ER, the SOD1 aggregates induced by tunicamycin did not colocalize with the mitochondria marker Mitotracker, with Tim17 which marks the mitochondrial inner membrane nor Tom20 which marks the mitochondrial outer membrane (Fig. 3J′–R′). The localization of these SOD1 aggregates also did not correspond with the Golgi apparatus or the lysosomes, which were stained by anti-GM130 antibody and Lyso-tracker, respectively (Fig. 3S′–X′).

**Figure 4:**
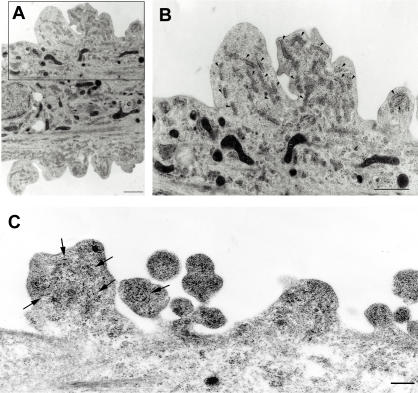
ER and SOD1 co-localization in peri-cytoplasmic membrane region. (A) Electron micrograph of L84V SOD1-expressing SK-N-SH cells after treatment with 1 µg/ml of tunicamycin for 24 h as described in Materials and Methods. (B) Enlargement of part of (A). Arrowheads indicate abnormal ER aggregates, where mutant SOD1 is localized as in Fig. 3C′ and 3E′. Scale bar = 1 µm. (C) SOD1 localization in peri-cytoplasmic membrane region. Cells were treated as described in (A) and immune electron micrograph was obtained as described in Materials and Methods. Arrows show SOD1 immunoreactive in ER.

Our previous results in figure 3C′, F′ and I′ revealed aberrant redistribution of ER membranes in tunicamycin-treated mutant SOD1 expressing cells to the cell periphery region. To directly visualize the localization of ER, we performed electron microscopic analysis of tunicamycin-stressed cells expressing mutant SOD1. Figure 4A and B showed abnormal aggregates of rough ER, sac-like structures with surface ribosomes, associated with numerous free ribosomes. Mutant SOD1 localization to these peripheral aggregates was confirmed by immunoelectron microscopy (Fig. 4C), implying defective functional activities of ER and free ribosomes in cells expressing mutant SOD1.

### LBHI/Ast-HI-like Inclusions are induced by ER stress

**Figure 5:**
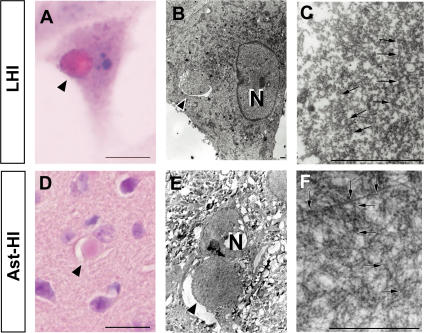
LHIs containing granule-coated fibrils are morphologically identical with Ast-HI from L84V transgenic mice. (A–F) Comparison of a LHI induced by ER stress in an L84V SOD1-expressing SK-N-SH cell (A–C) and Ast-HI in the spinal cord of a transgenic L84V SOD1 mouse (D–F). (A) An eosinophilic LHI in the cytoplasm of the SK-N-SH cell expressing L84V SOD1 cell was induced by treatment with 1 µg/ml of tunicamycin for 24 h (scale bar = 20 µm). (B) Electron micrograph of a hyaline inclusion (arrow) obtained by the direct epoxy resin-embedding method after decolorization of the HE-stained section shown in (A). N, nucleus; ×3000 (scale bar = 1 µm). (C) At a high magnification, the inclusion is composed of granule-coated fibrils (arrows) approximately 15–25 nm in diameter and granular materials. ×16000 (scale bar = 1 µm). (D) An eosinophilic Ast-HI from a transgenic L84V SOD1 mouse. (E) Electron micrograph of an Ast-HI obtained by the direct epoxy resin-embedding method mentioned in (B). N, nucleus; ×2000 (scale bar = 1 µm). (F) Enlargement of (E). ×16000 (scale bar = 1 µm). Note that the fibrils observed in (C) and (F) are ultrastructually identical.

Wate et al. [28] reported that neuronal LBHI in G93A SOD1 transgenic mice are immune reactive for GRP78/BiP, an ER resident component of the UPR response. As shown in figures 3A′-I′ and 4C, mutant SOD1 localized to the ER following stress induction by tunicamycin. These SOD1 aggregates shared additional features with LBHI/Ast-HI, namely eosin positivity and ubiquitin immune reactivity. Those observations led us to consider whether ER stress would eventually induce the formation of full-fledged LBHI/Ast-HI. To test this hypothesis, we examined whether inclusion bodies containing mutant SOD1 developed in L84V SOD1-expressing cells subjected to ER stress. Consistent with this idea, eosinophilic hyaline inclusions (∼10 to 20 µm in diameter) with a pale core, which are similar to neuronal LBHI/Ast-HI in the spinal cord of ALS patients harboring a SOD1 mutation, developed within 24 hrs of exposure to tunicamycin (Fig. 5A), but not in cells expressing wild type SOD1 (data not shown). In fact, the eosin-positive LBHI/Ast-HI-like hyaline inclusions (LHIs) were morphologically similar to the Ast-HI seen in the spinal cord of transgenic L84V SOD1 mice at the symptomatic stage (Fig. 5A and D). Furthermore, ultrastructual analysis revealed that the LHIs in neuroblastoma cells were composed of granule-coated fibrils (approximately 15–25 nm in diameter) and granular materials, which are the typical morphological hallmarks of mutant SOD1-linked FALS, and were identical with the Ast-HI found in L84V SOD1 mice (Fig. 5C, F; [38]). These results suggest that LBHI/Ast-HI in FALS patients might be provoked by ER stress as we observed for LHIs.

**Figure 6:**
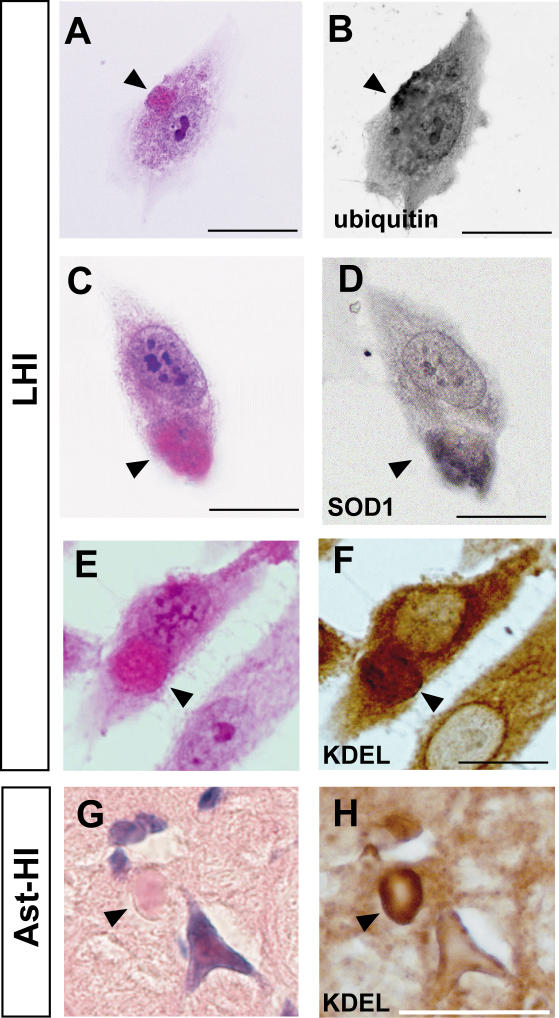
Positive immunoreactive against ubiquitin, SOD1 and KDEL of LHIs. (A–D) LHIs show immunoreactive against ubiquitin and SOD1. Eosinophilic LHIs in SK-N-SH cells (arrowheads in A and C) induced by tunicamycin were immunostained for ubiquitin (B) and SOD1 (D) after de-colorization. (E–H) KDEL immunoreactive in both LHI and Ast-HI. Eosinophilic LHI in SK-N-SH cells (arrowhead in E) and Ast-HI in spinal cord of L84V SOD1 mouse (arrowhead in G) were immunostained against anti-KDEL antibody after de-colorization (F, H). Scale bar = 20 µm

We further explored the molecular similarity between the LHI and LBHI/Ast-HI, using double-label immunocytochemistry. As shown in figure 6A–D, LHIs induced by tunicamycin are immunopositive for anti-SOD1 and anti-ubiquitin antibodies, consistent with the LBHI/Ast-HI features. In the spinal cord of G93A SOD1 mutant mice at the symptomatic stage, neuronal LBHI show GRP78/BiP immunoreactive, suggesting the involvement of ER resident protein [28]. Therefore, we examined whether LHIs also contain ER resident protein. As expected, LHI showed anti-KDEL positivity, indicating the involvement of ER resident proteins such as calreticulin, GRP 94, PDI and GRP78/BiP in LHI development (Fig. 6E and F). Furthermore, Ast-HI in spinal cord of L84V SOD1 transgenic mice at symptomatic stage also showed KDEL positive (Fig. 6G and H), meaning that the principle features of these inclusions in neuroblastoma cells and the LBHI/Ast-HI of FALS patients are the same and implying LHI and LBHI/Ast-HI might develop in similar procedure.

### Abnormal ER aggregated around peri-nuclear region with numerous free ribosomes at presymptomatic stage of Ast-HI in L84V SOD1 mice

**Figure 7:**
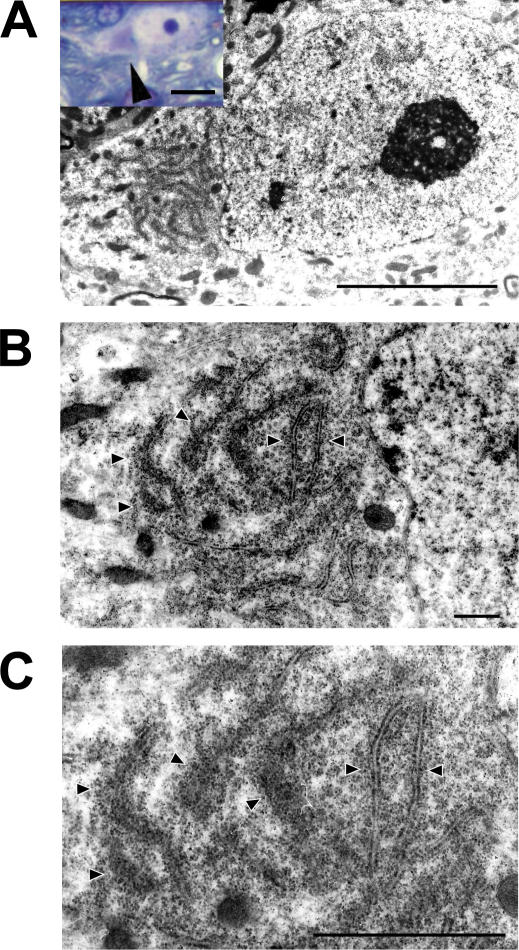
ER shows abnormal aggregation with numerous free ribosomes in L84V SOD1 mouse at presymptomatic stage. (A–C) Electron micrographs of a neuron obtained from an L84V SOD1 transgenic mouse containing ER aggregates. The inset in (A) shows a cytoplasmic inclusion-like structure (arrowhead) stained with toluidine blue. (A) ×3500 (scale bars = 20 µm). (B) ×8000 (scale bar = 1 µm). (C) ×15000 (scale bar = 1 µm). Arrowheads indicate abnormal ER aggregates.

To further explore the relationship of LHI to the development of LBHI/Ast-HI in FALS patients with mutant SOD1, we performed ultrastructual examination of transgenic L84V SOD1 mice, which show neuronal LBHI and Ast-HI at symptomatic stage (Fig. 5D–F, 6G–H; [35]). We examined the mice at the presymptomatic stage in the hope of detecting precursors to hyaline inclusion bodies. In spinal cord neurons of the presymptomatic L84V SOD1 transgenic mice, we observed aberrant aggregation of electron-dense rough ER around the peri-nuclear region with numerous free ribosomes, which were suspected to be producing mutant SOD1 (Fig. 7). This suggests that the aberrant SOD1 fibrils observed in spinal neurons of these mice at later stages might be produced by cooperative activity of ER and ribosomes. These inclusion-like structures with abnormal accumulation of ER seemed likely to represent a precursor to the later neuronal LBHI observed in this line. These results imply that the deterioration of ER function and the involvement of ER might be important for formation and developing neuronal LBHI/Ast-HI in mutant SOD1 harboring FALS patients.

## Discussion

Aggregated proteins or inclusions are a pathological hallmark and possible causative agent of several neurodegenerative disorders including ALS [39]. While LBHI/Ast-HI have been established as morphological hallmarks of mutant SOD1-linked FALS, little is known about the formation of these structures in neurons [6]. Several in vitro systems have been provided for analysis mutant SOD1 aggregation [35], [36], [40], however, the relationship between mutant SOD1 aggregation *in vitro* and pathological hyaline inclusions *in vivo* remains unclear. The LHI we observed in SK-N-SH cells expressing mutant SOD1 provide a direct link between *in vitro* and *in vivo* SOD1 aggregation. To our knowledge, this is the first study to show reproducible induction of LBHI/Ast-HI like structures meeting the criteria of inclusion bodies [24], [26], [31], [38], [41].

LBHIs/Ast-HIs in human FALS consist of a chaotic mixture of cytoplasmic proteins (such as SOD1, copper chaperone for SOD (CCS), peroxiredoxin 2, and glutathione peroxidase 1), cytoskeletal proteins (such as tubulin, tau protein, and phosphorylated- and nonphosphorylated neurofilament), nuclear proteins (such as neuron-specific enolase) and synaptic proteins (such as synaptophysin [24], [38], [41]–[43]). Recently, it has been published that GRP78/BiP, an ER resident chaperon protein, is also co-localized with LBHI of G93A SOD1 mice [28]. GRP78/BiP is molecular chaperone protein induced by IRE1 in response to aberrant protein folding and promotes proper protein folding. In this context, GRP78/BiP may be acting as part of the UPR response to resolve granule coated fibrils. Tobisawa et al. [35] reported increased protein levels of GRP78/BiP in motor neurons of mutant SOD1 transgenic mice, suggesting that the motor neurons in their model suffer from ‘ER stress’. While the importance of ER stress or proteosome malfunction in formation of mutant SOD1 aggregates has been established [35], [36], [40], the mechanisms by which mutant SOD1 forms LBHI/Ast-HI in FALS remain poorly understood. In this study, we present three lines of evidence for the involvement of ER stress in early events in LBHI/Ast-HI formation. First, ER stress in neuroblastoma cells expressing mutant SOD1 results in SOD1- and ubiquitin-immunopositive LHIs, compatible with LBHI/Ast-HI, composed of granule-coated fibrils approximately 15–25 nm in diameter and granular materials (Figs. 5 and 6). Secondly, we observed similar structures in the spinal cord of L84V SOD1 transgenic mice at presymptomatic stages, including abnormal electron dense, i.e. stressed, ER and numerous free ribosomes. (Figs. 4 and 7). Third, positive staining against anti-KDEL antibody, which recognizes ER resident proteins such as calreticulin, GRP 94, PDI and GRP78/BiP, were observed in both the LHI and Ast-HI of L84V SOD1 transgenic mice at symptomatic stages (Fig. 6E–H). These findings support the hypothesis that ER stress induces LBHIs/Ast-HIs creation in FALS patients with mutant SOD1. Taken together, these observations suggest that LHI in neuroblastoma cells and LBHI/Ast-HI in FALS patients might develop through similar processes.

In this study, we presented evidences that ER stress causes aggregates of mutant SOD1 and formation of LHI which is compatible with LBHI/Ast-HI. However, other questions arise from these results. 1) Why did same stress induce the different outcome of mutant SOD1 aggregation in the neuroblastoma? 2) Are the smaller aggregates competent to develop to LHIs? To answer these questions, we sought without success to identify the origin of the granule coated fibrils or SOD1 containing filamentous structure (e.g. less densely coated fibrils) in the smaller SOD1 aggregates localized to ER in L84V SOD1 expressing cells. Nevertheless, we found common features between the small aggregates in L84V SOD1 expressing SK-N-SH cells and neuronal LBHI-precursor in L84V transgenic mice, including regions of abnormal ER aggregation surrounded by abundant free ribosomes (Fig. 4B and Fig 7C). Furthermore, LHI and Ast-HI were immunopositive for the KDEL peptide present in ER-resident proteins, suggesting the involvement of ER itself in formation or development of LBHI/Ast-HI (Fig. 6E–H). We suggest that aberrant SOD1 fibril might be produced by cooperative activity of ER and ribosomes. To answer the questions, careful observation of LHI with time lapse analysis is needed.

It remains unclear why the major symptoms of ALS in patients with mutant SOD1-linked FALS do not develop until middle age, but we speculate that age-dependent changes in responses to ER stress might provide an answer. Under normal conditions, newly synthesized and misfolded proteins are refolded by chaperons such as GRP78, 94, calnexin, and calreticulin. This UPR response may be more robust in younger FALS patients and might be the reason the proteins aggregates are not observed in young patients even though mutant SOD1 is expressed. However, a decrease in protein folding or chaperone capability may occur with aging, and accumulation of misfolded proteins in the ER lumen may gradually lead to ER stress [44]. Consistent with this idea, Tobisawa et al. reported mutant SOD1 retention in the ER in COS7 cells [35] and Kikuchi et al. reported age-dependent increase of mutant SOD1 aggregation to ER in spinal cord of G93A SOD1 mice, suggesting ER dysfunction might be caused by mutant SOD1 [36]. Prolonged ER stress associated with insufficient degradation of misfolded proteins would subsequently activate apoptotic pathways. Nakagawa et al. reported that caspase-12, the ER resident caspase, is specifically cleaved and activated by ER stress, and that cells derived from mice lacking caspase-12 are resistant to ER stress [16]. In the spinal cords of G93A SOD1 mice, caspase-12 is activated in symptomatic period and can be inhibited by overexpression of XIAP (X-linked inhibitor of apoptosis protein [45], [46]. Then, we analyzed activation of caspase-4 (the human orthologue of rodent caspase-12) following tunicamycin treatment. As expected, the SOD1 aggregates of the L84V SOD1-expressing neuroblastoma cells colocalized with caspase-4 (unpublished data), implying caspase-4 might contribute to cell death in our model system.

Although it can take longer than 30 years for LBHI/Ast-HI to develop in FALS patients, we could induce the formation of morphologically similar LHI within 24 hours in our simple model. Detection of the molecular targets for ER stress-induced hyaline inclusions of mutant SOD1 in our model might lead to the development of therapy that can prevent the progression of mutant SOD1-linked FALS. Ultimately, our study should contribute to the development of a simple system to analyze novel therapies for ALS.

## Materials and Methods

### Transgenic Mice

Transgenic mice for mutant human SOD1^L84V^ (C587BL/6 background) were created (M. Kato, et al. Transgenic mice with ALS-linked SOD1 mutant L84V. Abstract of the 31st Annual Meeting of Society for Neuroscience, San Diego, 2001). Mice were genotyped by PCR to detect the mutant SOD1 transgene using the following primers: forward, TTGGGAGGAGGTAGTGATTA; reverse, GCTAGCAGGATAACAGATGA. The onset of symptoms was at 5–6 months and the initial sign of the disease was usually weakness in their hindlimbs, while approximately 10% of the mice first showed weakness in their forelimbs.

### Chemicals and antibodies

We used the following antibodies: anti-SOD1 polyclonal antibody (pAb; Chemicon, Temecula, CA); anti-ubiquitin pAb and anti-KDEL mAb (Stressgen, Victoria, BC. Canada); anti-Tim17 pAb and anti-Tom20 pAb (grateful gifts by Dr. Otera and Prof. Mihara [47], [48]); Alexa Fluor 488-conjugated anti-sheep IgG, Alexa Fluor 588-conjugated anti-mouse IgG antibody, and Alexa Fluor 588-conjugated anti-rabbit IgG antibody (Molecular Probes, Eugene OR); biotinylated anti-sheep IgG (Vector Laboratories, Burlingame, CA); anti-FLAG mAb (Sigma, woodlands, USA); anti-myc pAb and anti-GFP-mAb (Santa Cruz, Santa Cruz, CA); HRP-conjugated anti-sheep IgG (Jackson ImmunoResearch Laboratories Inc., West Grove, PA); and HRP-conjugated anti-mouse IgG and HRP-conjugated anti-rabbit IgG antibody (Cell Signaling Technology, Beverly, MA). Tunicamycin was obtained from Sigma.

### Cell culture and induction of ER stress

SK-N-SH human neuroblastoma cells were obtained from the Riken Cell Bank (Tsukuba, Japan), and were cultured in α-MEM (Invitrogen) containing 10% fetal bovine serum at 37°C under 5% CO_2_. These cells were transfected with pcDNA3.1-hSOD1 and pcDNA3.1-hL84V-SOD1 to cause overexpression of wild-type or L84V mutant SOD1, respectively. G418 resistant stable neuroblastoma cell lines expressing equal levels of endogenous and exogenous SOD1 were established. In all experiments, we used cultures that were at 70–80% confluence to avoid the influence of stress induced by overgrowth. On the day of stimulation, fresh medium was added more than 1 h before exposure to stress in order to ensure the same conditions for each culture.

### Western blot analysis

SK-N-SH cells stably expressing wild-type or L84V SOD1 were washed with PBS, harvested, and lysed in TNE buffer containing 1 mM PMSF and 1% SDS. 10 µg of protein was subjected to 12% SDS-PAGE and transferred to a PVDF membrane (Millipore Corp.). The membrane was blocked with 5% skim milk and incubated with anti-SOD1 antibody (1∶1500 dilution), followed by incubation with an HRP-conjugated secondary antibody. Proteins were visualized with an ECL detection system (Amersham-Pharmacia).

### Immunocytochemistry

SK-N-SH cells stably expressing wild-type SOD1 or L84V SOD1 were treated with 1 µg/ml of tunicamycin for 24 h. Then the cells were fixed with Zamboni's solution (0.1 M phosphate-buffered saline (PBS; pH 7.4) containing 2% paraformaldehyde (PFA) and 21% picric acid), rinsed in 0.1 M PBS, and incubated for 30 min in 0.3% H_2_O_2_ to eliminate endogenous peroxidases. Next, the cells were incubated overnight at 4°C with the primary antibody (a polyclonal sheep anti-SOD1 antibody; Calbiochem) at 1∶1000 in 0.1 M PBS containing 0.3% Triton X-100 and 3% bovine serum albumin (BSA). After washing in 0.1 M PBS, cells were incubated for 30 min with the secondary antibody (biotinylated anti-sheep IgG) (Vector Laboratories). After amplification with avidin-biotin complex from the ABC kit (Vector Laboratories), reaction products were visualized with 0.05 M Tris-HCl buffer (TBS; pH 7.6) containing 0.02% diaminobenzidine tetrahydrochloride (DAB) and 0.01% hydrogen peroxide. Finally, the cells were counterstained with Mayer's hematoxylin and eosin (HE).

### Co-immunoprecipitation assay utilizing ubiquitin

Lysates of pcDNA3.1-myc-tagged ubiquitin (a kind gift from Dr. Niwa and Prof. Sobue [32])-transfected SK-N-SH cells stably expressing wild-type SOD1 or L84V SOD1 were prepared using TNE buffer (10 mM Tris-HCl, (pH 7.4), 150 mM NaCl, and 1 mM EDTA) containing 1 mM phenylmethylsulphonyl fluoride (PMSF), 2 µg/ml aprotinin, and 1% Nonidet P-40 after treatment with or without 4 µg/ml ALLN for 12 h. Then, 1 µg of anti-FLAG antibody was added to 400 µg of lysate, followed by incubation at 4°C for at least 3 h. Protein G-Sepharose (10 µl gel) was then added and incubation was done with rotation at 4°C for 1 h. The immunoprecipitate was subjected to SDS-PAGE and transferred to a polyvinylidene fluoride (PVDF) membrane. The membrane was blocked with 5% skim milk and then was incubated with anti-Myc antibody (1∶1000 dilution), followed by incubation with an HRP-conjugated secondary antibody. Proteins were visualized with an ECL detection system (Amersham-Pharmacia).

### Immunofluorescence and chemifluorescence

SK-N-SH cells expressing wild-type SOD1 or L84V SOD1 were incubated with or without tunicamycin or ALLN, rinsed in 0.02 M PBS, and fixed in Zamboni's fixative. Then the cells were incubated overnight at 4°C with an anti-SOD1 antibody (1∶1000 dilution) and either anti-KDEL (1∶500 dilution), anti-GM130 (1∶500 dilution) or anti-ubiquitin (1∶500 dilution) antibody in 0.02 M PBS containing 0.3% Triton X-100 and 3% BSA. Next, the cells were treated with fluorescent dye (Alexa Fluor 488)-conjugated donkey anti-sheep IgG (SOD1; 1∶1000 dilution), fluorescent dye (Alexa Fluor 568)-conjugated goat anti-mouse IgG (KDEL, GM130; 1∶1000 dilution), and goat anti-rabbit IgG (ubiquitin; 1∶1000) as the secondary antibodies for 1 h at RT in 0.02 M PBS containing 3% BSA. Examination was done under a Zeiss LSM 510 microscope. For detection of SOD1 colocalization with cytochrome b5, pCMV b5-EGFP vector was transfected to the cells (kind gift from Dr. Otera and Prof. Mihara; [49]). The GFP signal was enhanced by anti-GFP antibody staining (1∶100). In order to determine the localization of SOD1 in living cells, SK-N-SH cells expressing wt and L84V SOD1 were transfected with a pcDNA3.1-GFP-tagged wt and L84V SOD1 plasmid, respectively. After treatment with tunicamycin for 24 hr, the cells were further incubated with Mito-tracker or Lyso-tracker (Molecular Probes) for 30 min to visualize the mitochondria or lysosomes, respectively. Then the cells were rinsed at least three times in 0.1 M PBS and fixed with Zamboni's solution for examination under a LSM 510 confocal microscope (Zeiss, Osaka, Japan).

### Electron microscopy

SK-N-SH cells stably expressing L84V SOD1 were exposed to 1 µg/ml tunicamycin for 24 h and then fixed at room temperature (RT) for 1 h in 0.1 M phosphate buffer (PB) containing 2.5% glutaraldehyde (GA) and 2% paraformaldehyde. Subsequently, the cells were post-fixed in 1% OsO_4_ at RT for 1 h, dehydrated in a graded ethanol series, and embedded in epoxy resin (Quetol 812; Nisshin EM Co.). Areas containing cells with aggregates were block-mounted in epoxy resin by the direct epoxy-resin embedding method and cut into 90-nm sections. The sections were counterstained with uranyl acetate and lead citrate, and then examined using an H-7100 electron microscope (Hitachi).

### Immune Electron microscopy

As with immunocytocemistry methods above, after fixation with Zamboni solution containing 0.1% GA, the cells with anti-SOD1 antibody were developed with DAB. Then, they were post-fixed in 1% OsO4 in 0.1 M PB at RT for 30 min after 1% GA in 0.1M PB re-fixation. The samples were dehydrated in a graded ethanol series and then embedded in Quetol 812. Areas containing cells with aggregate morphology were block-mounted and cut into 90-nm sections. The sections were counterstained with uranyl acetate and lead citrate, and then examined with an H-7100 electron microscope.

### Analysis of inclusion bodies (light microscopy and electron microscopy)

Sections of SK-N-SH cells containing eosinophilic hyaline inclusion bodies and spinal cord sections from transgenic SOD1 L84V mice were decolorized, rehydrated, rinsed in 0.1 M PBS, and then blocked for 1 h in 0.1 M PBS containing 0.3% Triton X-100 and 3% BSA. Next, the sections were incubated overnight at 4°C with the primary antibody (polyclonal sheep anti-SOD1 antibody at 1∶500) in 0.1 M PBS containing 0.3% Triton X-100 and 3% BSA. After washing in 0.1 M PBS, sections were incubated for 30 min with the secondary antibody (biotinylated anti-sheep IgG). Subsequently, incubation was performed for 30 min in 3% H_2_O_2_ to eliminate endogenous peroxidases. After amplification with avidin-biotin complex (ABC kit, Vector Laboratories), visualization of reaction products was done with 0.05 M TBS (pH 7.6) containing 1.25% DAB and 0.75% hydrogen peroxide.

For electron microscopy, samples of SK-N-SH cells expressing L84V SOD1 and spinal cords from transgenic SOD1 L84V mice were decolorized, rehydrated, and rinsed in 0.1 M PBS. The samples were further fixed and dehydrated. Then the samples were embedded directly in epoxy resin, sectioned, counterstained, and examined as described under electron microscopy section.

## Supporting Information

Figure S1 Cytosolic localization of SOD1 in wt SOD1 expressing cells under ER stress. (A-F, A′-F′) Analysis of localization of SOD1 on ER. WT SOD1-expressing SK-N-SH cells were incubated for 24 h without (A-F) or with 1 ug/ml of tunicamycin (A′-F′). Then the cells were fixed and stained using an anti-SOD1 antibody (green; A, D, A′, D′) and an anti-KDEL antibody (red; B, B′) or an anti-GRP78 antibody (red; E, E′). GFP-cytochrome b5 were transfected to the cells and stained with anti-GFP (green; G, G′) and anti-SOD1 (red; H, H′) antibodies. Merged images (C, F, I, C′ F′, I′). (J-R, J′-R′) Analysis of SOD1 localization to the mitochondria. WT SOD1-expressing SK-N-SH cells were treated as described in above. The locations of the mitochondria and SOD1 were visualized in WT SOD1-expressing SK-N-SH cells using 100 nM Mito-tracker (red; K, K′), an anti-Tim17 antibody (red; N, N′) or an anti-Tom20 antibody (red; Q, Q′) and an anti-SOD1 antibody (green; J, M, P, J′, M′, P′). Merged images (L, O, R, L′, O′, R′). (S-U, S′-U′) Investigation of SOD1 localization to the Golgi apparatus. L84V SOD1-expressing SK-N-SH cells were treated as described in above. Then the cells were stained with anti-SOD1 antibody (green; S, S′) and anti-GM130 antibody (red; T, T′). Merged images (U, U′). (V-X, V′-X′) Analysis of the localization of SOD1 to the lysosomes. A GFP-tagged WT SOD1 vector was transfected into WT SOD1-expressing SK-N-SH cells. After 24 h of incubation with 1 ug/ml of tunicamycin, the cells were incubated for a further 30 min with 100 nM Lyso-tracker (red; W, W′) to visualize the lysosomes. GFP channel (V, V′) Merged images (X, X′). Scale bars = 20 um.
(3.70 MB TIF)
