# Supplementary figures and images for: An In Vitro Model for Lewy Body-Like Hyaline Inclusion/Astrocytic Hyaline Inclusion: Induction by ER Stress with an ALS-Linked SOD1 Mutation
Source: PLoS One. 2007 Oct 10;2(10):e1030. doi: 10.1371/journal.pone.0001030 (PMC2000355; doi:10.1371/journal.pone.0001030)

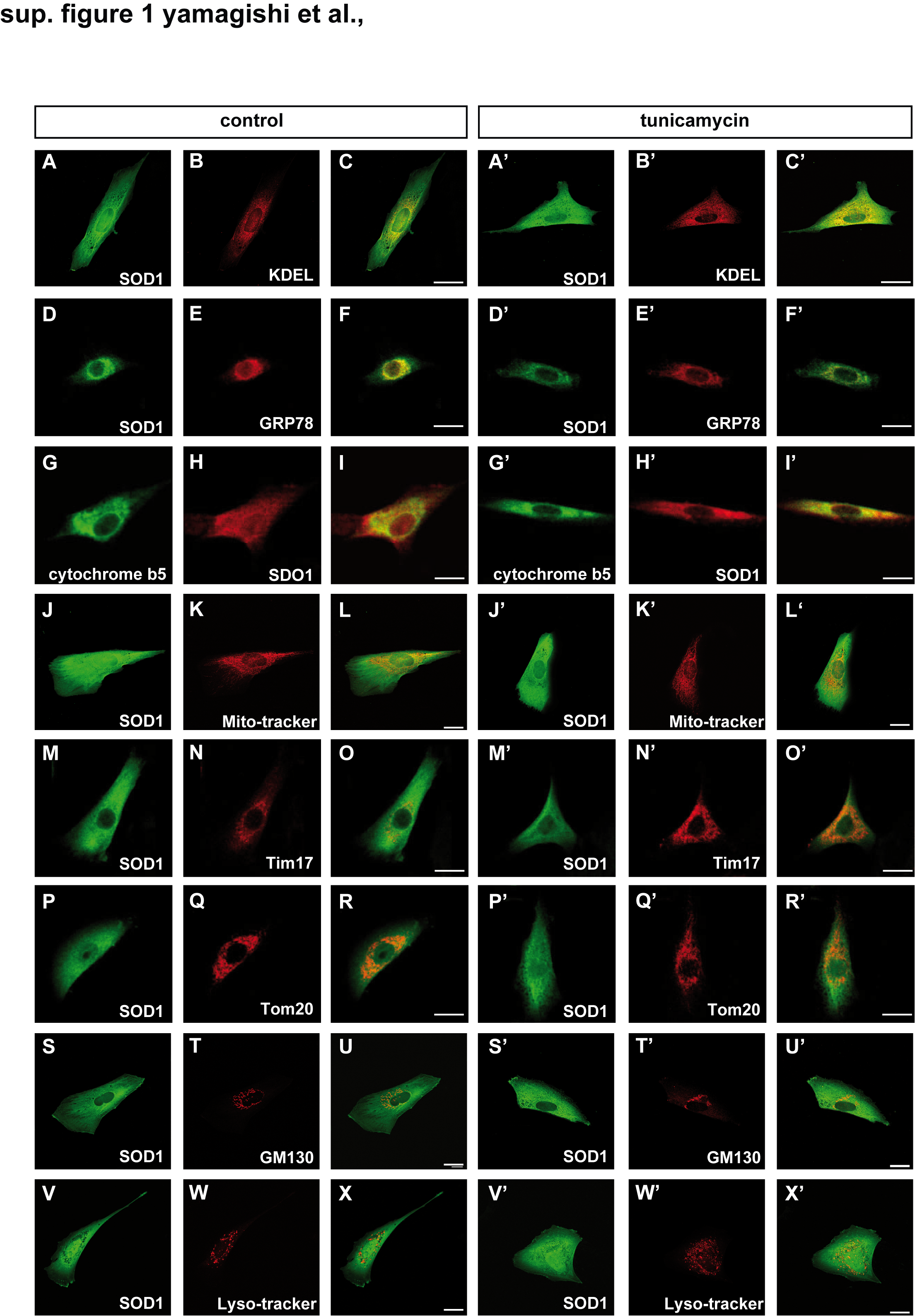

Supplement: Figure S1 — Cytosolic localization of SOD1 in wt SOD1 expressing cells under ER stress. (A-F, A′-F′) Analysis of localization of SOD1 on ER. WT SOD1-expressing SK-N-SH cells were incubated for 24 h without (A-F) or with 1 ug/ml of tunicamycin (A′-F′). Then the cells were fixed and stained using an anti-SOD1 antibody (green; A, D, A′, D′) and an anti-KDEL antibody (red; B, B′) or an anti-GRP78 antibody (red; E, E′). GFP-cytochrome b5 were transfected to the cells and stained with anti-GFP (green; G, G′) and anti-SOD1 (red; H, H′) antibodies. Merged images (C, F, I, C′ F′, I′). (J-R, J′-R′) Analysis of SOD1 localization to the mitochondria. WT SOD1-expressing SK-N-SH cells were treated as described in above. The locations of the mitochondria and SOD1 were visualized in WT SOD1-expressing SK-N-SH cells using 100 nM Mito-tracker (red; K, K′), an anti-Tim17 antibody (red; N, N′) or an anti-Tom20 antibody (red; Q, Q′) and an anti-SOD1 antibody (green; J, M, P, J′, M′, P′). Merged images (L, O, R, L′, O′, R′). (S-U, S′-U′) Investigation of SOD1 localization to the Golgi apparatus. L84V SOD1-expressing SK-N-SH cells were treated as described in above. Then the cells were stained with anti-SOD1 antibody (green; S, S′) and anti-GM130 antibody (red; T, T′). Merged images (U, U′). (V-X, V′-X′) Analysis of the localization of SOD1 to the lysosomes. A GFP-tagged WT SOD1 vector was transfected into WT SOD1-expressing SK-N-SH cells. After 24 h of incubation with 1 ug/ml of tunicamycin, the cells were incubated for a further 30 min with 100 nM Lyso-tracker (red; W, W′) to visualize the lysosomes. GFP channel (V, V′) Merged images (X, X′). Scale bars = 20 um. (3.70 MB TIF) [file pone.0001030.s001.tif]
